# Supplementary material for: Computing-in-memory architecture for Kolmogorov-Arnold networks based on tunable Gaussian-like memory cells
Source: Nat Commun. 2026 Feb 20;17:3496. doi: 10.1038/s41467-026-69592-w (PMC13079891; doi:10.1038/s41467-026-69592-w)
Supplement: Supplementary file 1 — Supplementary Information [file 41467_2026_69592_MOESM1_ESM.pdf]

Supplementary Information for

Computing-In-Memory Architecture for Kolmogorov-Arnold Networks Based on Tunable Gaussian-like Memory Cells

Zhixing Wen[1,2,†], Qirui Zhang[1,†], Jiangang Chen[1,†], Tianhua Yang[1], Fan Yang[1], Xuemei Wang[1], Qing Liu[1], Xiao Luo[1,*], Peng Lin[3], Liang-Jian Deng[4,*], Fucai Liu[1,5,*]

1 School of Optoelectronic Science and Engineering, University of Electronic Science and Technology of China, Chengdu 611731, China.

2 Yangtze Delta Region Institute (Huzhou), University of Electronic Science and Technology of China, Huzhou 313001, China.

3 College of Computer Science and Technology, Zhejiang University, Hangzhou 310058, China.

4 School of Mathematical Sciences & Multi-Hazard Early Warning Key Laboratory of Sichuan Province, University of Electronic Science and Technology of China, Chengdu 611731, China.

5 State Key Laboratory of Electronic Thin Films and Integrated Devices, University of Electronic Science and Technology of China, Chengdu 611731, China.

† These authors contributed equally to the work.

* E-mail: luox@uestc.edu.cn (X.L.), liangjian.deng@uestc.edu.cn (L.D.), fucailiu@uestc.edu.cn (F.L.).

Supplementary Notes

Supplementary Note 1: A comprehensive analysis of computational paradigms for executing KAN's operations across various hardware implementations.

Gaussian kernels (simplified expression of Gaussian-like basis functions) serve as the fundamental computational elements within KANs, and their effective realization in hardware is pivotal for the practical deployment of such networks. The feasibility of implementing and training these kernels in hardware or circuits is a critical factor in determining whether KANs can achieve their full potential. However, traditional hardware architectures, including von Neumann and near-memory systems, encounter considerable limitations in both the computation and training of Gaussian kernels, which impedes the realization of KANs' capabilities. In this context, GMCs have been introduced as an effective solution to address the dilemmas that existing devices and architectures are unable to overcome (as summarized in detail in Fig. 2 of the main text). To emphasize the transformative potential of GMC-based implementations for hardware KANs, the subsequent content will provide concrete examples that illustrate the shortcomings of conventional hardware approaches, thereby underscoring the advantages of G-KANs.

(1) Dilemmas of von Neumann architecture (corresponding to Dilemma 1-3 in Fig. 2b of the main text)

In the von Neumann architecture, the physical separation of the processor and memory necessitates continuous data and instruction transfers during execution. This results in a significant mismatch between memory bandwidth and processing

throughout, often leaving CPU and GPU cores underutilized as they wait for data. Furthermore, accessing memory incurs substantial time and energy costs, with much of the energy spent on data transfer rather than actual computation (Dilemma 1 and 2).[1,2] The graphics processing unit (GPU), a representative example of optimized computing, has gained widespread adoption due to its remarkable acceleration of general matrix multiplication (GEMM)[3]. GPUs excel at performing linear transformations with high computational density by taking advantage of regular operations and contiguous memory access[4], while the central processing unit (CPU) only needs to initiate a single scheduling process, minimizing offloading overhead.[5] However, these advantages are not fully realized in KANs' computation. Using Gaussian kernels as basis functions, the inference process involves sequential operations such as squaring ($(x-a)^2$), scaling multiplication ($-\frac{1}{\sigma}\cdot(x-a)^2$), nonlinear exponential transformation ($\exp(\cdot)$), weighted multiplication ($c_i\cdot\exp(\cdot)$), and accumulation ($\sum_i$). These operations cannot be directly handled by GEMM, requiring frequent kernel launches per feedforward pass (each scheduled by the CPU), which significantly increases CPU-GPU offloading hardware resources and power than MLPs with the same number of parameters.[2,6]

(2) Dilemmas of near-memory implementations (corresponding to Dilemma 4-6 in Fig. 2b of the main text)

The evidence above confirms the incompatibility of KANs with the von Neumann architecture. Therefore, it is crucial to implement KANs on hardware platforms with higher computational and memory density. In this context, researchers have attempted to alleviate the dilemmas of von Neumann architecture through near-memory

computing[7]. For KAN-related applications, typical examples include lookup table (LUT)[8] and piecewise linear (PWL)[9] circuit designs. Although LUT-based circuits can directly output Gaussian kernels at the hardware level, this strategy inevitably introduces significant computational complexity and storage address overhead. For instance, implementing a single Gaussian kernel requires 452 LUTs.[8] When scaled to a network level, for highly densely integrated architectures like KANs, the resulting operational costs become unbearable (Dilemma 4).

Moreover, a recent study have employed CMOS integration technology based on PWL to directly approximate the activation functions (connections) in KANs.[9] However, this strategy has several inherent bottlenecks: first, the PWL function requires predefined breakpoint (X_{n}), slope (M_{n}) and intercept (Y_{n}) of each segment ($X_{\mathrm{n}-1} \leq X < X_{\mathrm{n}}$), which must be stored in peripheral memories; second, the hardware architecture must integrate massive comparators (COMPs) and sensing circuits to determine which segment the input signal belongs to, then retrieve the corresponding slope and intercept from the buffers and pass these parameters to the MAC circuits to complete the linear operation ($F(X) = M_{\mathrm{n}} * X + Y_{\mathrm{n}}$). Therefore, even for simple and few target operations, this approach still suffers from heavy hardware resource overhead (Dilemma 5).

Overall evaluation of these two strategies reveals that they not only require significant hardware resources such as multipliers (MPLs), adders (ADDs), and COMPs to execute Gaussian kernel computations, but also, due to their hardwired electrical architecture, are physically incapable of implementing parameter update

functionality (Dilemma 6). Therefore, although both aim to alleviate the von Neumann bottleneck, LUT-based and PWL-based implementations ultimately suffer from a fundamental flaw due to their inability to endow the neural network with trainable characteristics.

In the context given above, the limitations of existing CMOS-based implementations have led to the exploration of dedicated KAN-type computing-in-memory (CIM) architectures. Clearly, memristors alone cannot achieve in-memory computation for nonlinear functions such as Gaussian kernels. The following content will specifically elaborate on how the GMCs and the array design proposed in this work effectively address the aforementioned challenges.

(3) Solution relying on GMC-based implementation (corresponding to the Solution 4-6 in Fig. 2b of the main text)

The transfer characteristics of GMCs can directly generate stable and parameterized Gaussian kernels, naturally representing the mathematical behavior of Gaussian kernels in the analog domain. In addition, by adjusting the conductance of memristors through write voltages, the peak height of the Gaussian kernel can be flexibly controlled, enabling electrical programming of the learnable coefficients. At the architectural level, the one-transistor-one-memristor structure of GMCs facilitates easy integration into crossbar arrays.[10] According to the KCL, the output currents of all GMCs in the same column can be physically accumulated on the source line, directly performing the linear combination of Gaussian kernels in a KAN. Therefore, the GMC-based implementation can complete the full computation from input to output within the analog domain

(Solution 1).

Furthermore, the Dilemma 2 in conventional CMOS-based implementations arises from the physical separation between processor and memory.[1] The GMC-based implementation fundamentally reshapes this paradigm through the CIM approach. The core mechanism lies in synchronizing computation and data storage at the same physical location, thus completely avoiding the frequent data transfers between memory and processing units. As a result, the conflict between memory bandwidth and processing throughput is eliminated, with energy consumption focused on computation (Solution 2). Meanwhile, the in-situ CIM working mode also entirely avoids the offloading overhead inherent in von Neumann architecture (Solution 3).

To address the hardware catastrophe scaling problem (Dilemma 4) faced by CMOS-based implementations—an inherent bottleneck where the growth in the number of basis functions triggers a nonlinear explosion in hardware resource consumption—the GMCs proposed in this study offers a fundamental solution through a linear hardware scaling paradigm. Specifically, under the differential-pair encoding rule, each Gaussian kernel requires only a fixed pair of GMCs. This ensures that the hardware scale expands strictly linearly with the number of Gaussian kernels, which is equivalent to the network scale of a KAN. As a result, this approach fundamentally prevents uncontrollable resource consumption at the hardware level, ensuring that the hardware scale of a G-KAN remains predictable and manageable throughout (Solution 4).

Of equal significance, the GMC-based paradigm circumvents the complex and

segmented operations that characterize near-memory computing implementations (Dilemma 5 and 6). Within the GMC arrays, an input vector is loaded in parallel across all rows, enabling each cell to perform in-situ Gaussian-like mapping and MAC operations simultaneously. By leveraging the native physics of the crossbar array and KCL, the complete matrix transformation is executed in one step, thereby obviating the need for piecewise function evaluation, parameter retrieval from memory, and data shuttling. This one-shot process fundamentally eliminates the timing and control overhead associated with multi-step operations in CMOS-based implementations, achieving a drastic reduction in computational complexity (Solution 5).

The primary dilemmas confronting the aforementioned CMOS-based implementations and their corresponding solutions enabled by the GMC-based implementations are graphically summarized in Fig. 2 of the main text. Furthermore, a detailed comparison of the key attributes discussed for the reference objects is provided in Supplementary Table 1.

Supplementary Table 1 Comparison for implementing KAN across different benchmarks.

Reference objects/ benchmarks	Von Neumann Architecture	Near-Memory Architecture	GMC-based Crossbar Array
Parameterized Gaussian kernel implementation	1. hardware dependance: DSPs/GPUs/FFs/ RAMs/LUTs[6,11,12]; 2. Muti-step pointwise operators (squaring→ multiplication→ exp()→ multiplication);	1. memory-bound; 2. Separation of parameterization and kernel computation; 3. Non-adjustable functions (hardwired); 4. Reliance on a large number of	physically in-device generation

Supplementary Table 1 (continued)

Reference objects/ benchmarks	Von Neumann Architecture	Near-Memory Architecture	GMC-based Crossbar Array
	3. memory-bound;[13,14]<br>4. frequent memory accessing[15]<br>☹️	computational modules[8,9]<br>☹️	😊
if one-shot?	No	No	Yes
Computing in memory	NOT<br>☹️	partial<br>☹️	complete<br>😊
if one-shot?	No	No	Yes
Pipeline	1. Data loading and scheduling initialization (CPU side);<br>2. Multi-step chain of pointwise operators;<br>3. Weighted accumulation;<br>4. Memory copy<br>☹️	(LUT-based)[8] ADC→multi-bit input (address)→ multi-bit output→ DAC;<br>(PWL-based)[9] binary encoding→ breakpoint detection →accessing slope/intercept memory→ parameter fetching →digital MAC→ DAC<br>☹️	analog MAC implemented on the crossbar array<br>😊
if one-shot?	No	No	Yes
Computational Parallelism	(partially parallel on GPUs)<br>1. memory-bound;[16]<br>2. high overhead of data movement;[17]<br>3. significant CPU-GPU offloading latency and energy consumption;[18]	(partially parallel)[9]<br>1. memory-bound;<br>2. massive computational modules;<br>3. intricated control flow (PWL-based scene);<br>4. redundancy in address (LUT-based	(completely parallel)<br>1. access-free memory;<br>2. offloading-free;<br>3. one-shot parallel VMM and MAC process;

Supplementary Table 1 (continued)

Reference objects/ benchmarks	Von Neumann Architecture	Near-Memory Architecture	GMC-based Crossbar Array
	4. high overhead of scheduling and synchronization[19,20]<br>☹️	scene) or parameter (PWL-based scene) access<br>☹️	4. simple control mechanism (voltage-pulse direct drive)<br>😊
Hardware overhead change	1. memory and computation demand grows superlinearly with the number of parameters;<br>2. exponentially increasing memory copy latency;*<br>3. memory bandwidth bottleneck amplifies with increasing computational scale[21]<br>☹️	1. memory and computation demand grows superlinearly with the number of parameters<br>☹️	1. device count demand increases linearly with computational scale (one basis function is implemented by a pair of GMCs)<br>😊
In-situ training	--	NO (hardwired)<br>☹️	YES<br>😊

* https://developer.nvidia.com/gdrcopy

Supplementary Note 2: Operating mechanism of the G-KAN circuit.

The electrical design of the G-KAN with additional residual connections is illustrated in Supplementary Fig. 9. The following content gives a detailed explanation about the working principle of the circuit from input to output in sequence for the entire workflow.

Given that the n_c basis functions are uniformly distributed, which can be achieved by equally spacing their centers into n_c positions, as illustrated in Supplementary Fig. 10. Given that the device transfer characteristic is described by $f(V_{\mathrm{g}}) = \exp\left[-\left(\frac{V_{\mathrm{g}} - V_{\mu}}{\sigma}\right)^2\right]$, and assuming the target basis function is expressed as $g(u) = \exp\left[-\left(\frac{u - V_{\mu}}{\sigma / \mathrm{k}}\right)^2\right]$. Herein, k is a positive parameter. The larger the value of k, the flatter the Gaussian kernel's bell-shaped distribution; conversely, the smaller the value of k, the shaper the bell-shaped distribution. As shown in Supplementary Fig. 10b, when transitioning from a coarse grid to a finer grid, it is necessary to correspondingly reduce the standard deviation of the Gaussian kernel (i.e., increase the value of k).[22] k serves as a topological hyperparameter, allowing the network to flexibly adjust the grid resolution. Based on the coordinate transformation $f\left(V_{\mathrm{g}}\right) = g\left(u\right)$, the following relationship is derived:

$$V_{\mathrm{g}} = \mathrm{k}u - (\mathrm{k} - 1)V_{\mu} \tag{S1}$$

Define $h(x) = \exp\left(-\frac{\mathrm{k}^2 x^2}{\sigma^2}\right)$. It is evident that $h(x + \Delta x) = \exp\left(-\frac{\mathrm{k}^2 (x + \Delta x)^2}{\sigma^2}\right)$. Let $g\left(u\right) = h\left(x + \Delta x\right)$, then the result follows that $u = x + \Delta x + V_{\mu}$. Therefore,

$V_g = k(x + \Delta x) + V_\mu$. Since the translated centers of $h(x)$ are evenly distributed across L points within the interval [-1,1], the set of Δx can be calculated as:

$$\Delta x \in \{1, 1-\delta, 1-2\delta, \ldots, -1+2\delta, -1+\delta, -1\} \; (\delta = \frac{2}{L-1}) \tag{S2}$$

In summary, the voltage sequence applied to the gates of different GMCs, according to the input signal (x), is given by that:

$$V_g \in \{k(x+1)+V_\mu, k(x+1-\delta)+V_\mu, \ldots, k(x-1+\delta)+V_\mu, k(x-1)+V_\mu\} \tag{S3}$$

In this design, the $b(x)$ array is integrated using a 1T1R structure to prevent miswriting during training.[10] For the signal routing, equipotential paths are employed with configurable interface switches to specify the desired connection combinations. During backpropagation, the potentials coordinate with the selected cells and the write voltage pulses to determine the target cell locations and the polarity of the pulses (SET or RESET). In array techniques, signal routing can be configured utilizing field-programmable gate arrays (FPGAs) in conjunction with peripheral circuits.[23]

Furthermore, the design of sensing electronics for the output stage is as follows. In the main text, the 'Subtractor Configuration' contains the peripheral circuits utilized to handle the differential operation of the output voltages, which can be implemented using an operational amplifier (op-amp) (Supplementary Fig. 11). Specifically, two transimpedance amplifiers (TIAs) as a differential pair convert the current representing the MAC operation result on the SL column into voltage, and then perform the differential calculation through an op-amp. The electrical principles in Supplementary Fig. 11 can be found in following description.

For the non-inverting terminal, according to the op-amp's virtual open and KCL,

the result follows that:

$$\frac{V_2 - V_+}{R_1} = \frac{V_+ - 0}{R_2} \Rightarrow V_+ = \frac{V_2}{1 + \frac{R_1}{R_2}} \tag{S4}$$

Similarly, for the inverting terminal, the result can be derived that:

$$\frac{V_1 - V_-}{R_1} = \frac{V_- - V_o}{R_2} \Rightarrow V_- = \frac{V_1 + \frac{R_1}{R_2} V_o}{1 + \frac{R_1}{R_2}} \tag{S5}$$

By also considering the virtual short characteristic ($V_+ = V_-$) of the op-amp, and combining Eq. S4 and S5, the final output (V_o) is exactly the result of the differential operation: $V_o = \frac{R_2}{R_1}(V_2 - V_1)$.

Based on the above design and circuit equivalence, a complete G-KAN's circuit (including the input voltage module, GMC array, and output sensing electronics) is constructed on the Simscape Electrical simulation platform (Supplementary Fig. 13).

Supplementary Note 3: Array scalability analysis

Given the intrinsic self-rectifying characteristics of the GMC devices, the read margin as the key metric to characterize the scalability of the GMC array[24]. The read margin reflects the ability of a memory array to accurately retrieve stored information under realistic conditions, including noise and sneak path currents. Therefore, it is a widely accepted and fundamental indicator for evaluating the scalability of self-rectifying memristive crossbar arrays. Additionally, the read voltage scheme during array operation has a significant impact on the sneak path current. The $V_{\text{read}}/2$ bias scheme is employed for current readout in this analysis. Under this scheme, only the fully selected GMCs (with a read voltage of V_{read}) will have current, while the partially selected (with a read voltage of $V_{\text{read}}/2$) and unselected GMCs (with a read voltage of 0) will have no current, effectively suppressing sneak-path currents.

Notably, the read margin typically decreases with array size, and a margin below 10% is generally considered the practical scalability limit.[25] Experimentally, we evaluate the read margin using a single bit-line pull-up strategy, which is defined by the following relationships[26]:

$$\text{Read Margin} = \frac{\Delta V}{V_{\text{pu}}} = R_{\text{pu}} \times \left(\frac{1}{R_1 + R_{\text{pu}}} - \frac{1}{R_2 + R_{\text{pu}}} \right) \tag{S6}$$

$$\begin{cases} R_{\text{sneak}} = \dfrac{2R_{\text{pos}}}{n-1} + \dfrac{R_{\text{neg}}}{(n-1)^2} \\ R_1 = R_{\text{LRS}} \times \left(\dfrac{R_{\text{sneak}}}{R_{\text{LRS}} + R_{\text{sneak}}} \right) \\ R_2 = R_{\text{HRS}} \times \left(\dfrac{R_{\text{sneak}}}{R_{\text{HRS}} + R_{\text{sneak}}} \right) \end{cases} \tag{S7}$$

Herein, ΔV refers to the difference between the input voltages of the selected cell at low-resistance state (LRS) and high-resistance state (HRS). V_{pu} denotes the pull-up voltage applied to the selected BL, which is represented by V_{read} in this experiment. R_{pu} is the pull-up resistance (the value is equal to the resistance at LRS in this experiment), and R_{sneak} is the resistance along the sneak paths. In addition, R_{pos} and R_{neg} refer to the resistance of a cell that is half selected in this experiment as well as a cell that is not selected at all, respectively. n represents the number of SLs or BLs in a whole crossbar array.

Supplementary Note 4: Expandable network property of residual connections

In addition to the combination of basis functions on each edge of a KAN, an extra trainable residual connection can also be added (Supplementary Fig. 12).[22] In this work, this connection is activated using a rectified linear unit (ReLU)[27], with the mathematical form given as:

$$b(x) = w_{\mathrm{b}}\mathrm{ReLU}(x) \tag{S8}$$

where the $\mathrm{ReLU}(x) = \max\{0, x\}$, and w_{b} is a synaptic weight involved in training.

Supplementary Note 5: Further discussion on whether task correlation and parameter redundancy contribute to continual learning in the 1D function regression tasks.

To strengthen the evidence that G-KAN maintains continual learning capability in the 1D function regression task, it is necessary to experimentally eliminate the influence of task correlation or potential parameter redundancy, thereby making the original conclusion more convincing.

(1) Parameter redundancy

G-KAN has more parameters than M-MLP in the same topologic architecture.[2] Therefore, if M-MLP, even with the identical or even more training parameters, still fails to achieve continual learning like KANs, this would help rule out the concern about parameter redundancy. As depicted in Supplementary Fig. 17, since the G-KAN was configured with 100 basis functions, the M-MLP initially preset a size of [1, 100, 1] to match the parameter count of both models. However, as clearly observed in Supplementary Fig. 17b, the M-MLP failed to achieve even basic fitting, let alone continual learning. Furthermore, after expanding the depth of the M-MLP sufficiently, the well-formed fitting Gaussian functions can be finally observed. Regrettably, each time the model learned a new Gaussian peak, it forgot all previous peaks, demonstrating that the M-MLP completely lacks the capability for continual learning in 1D function regression scenarios (Supplementary Fig. 17c). These results further solidify the recognition that KANs, with their local activation capabilities, inherently possess continual learning as a functional attribute, in contrast to MLPs, which rely on global

activation.

(2) Task correlation

In the main manuscript, each function period in a dataset was obtained via translation, and all periods consisted entirely of Gaussian functions. To mitigate potential concerns regarding task correlation, it is necessary to ensure the data within different periods do not completely overlap, and also introduce datasets generated by other functions. Based on this approach, function regression performed on this basis can validate whether the continual learning capability of G-KANs is independent of task correlation. This scenario encompasses three cases: non-Gaussian periodic, non-periodic, and piecewise functions.

Specifically, Supplementary Fig. 14a presents a periodic function, but since each half wave is treated as a separate subset, the training samples in each period do not completely overlap. In contrast, Supplementary Fig. 14b presents a non-periodic function, under which G-KAN continues to exhibit strong continual learning capability. Supplementary Fig. 14c represents a more challenging case, in which the G-KAN is trained on a piecewise function with intentionally introduced discontinuities between segments to minimize task correlation. As illustrated in this subplot, even when presented with unrelated sequential data, the G-KAN still demonstrates effective continual learning performance. Apart from a slight impact near the discontinuous boundaries, no significant catastrophic forgetting can be observed throughout the whole process. From the three subplots in Supplementary Fig. 14, it is clearly observed that whether the dataset consist of periodic, non-periodic, or piecewise functions, G-KANs

can perfectly fit the ideal function curves. This set of experiments demonstrated that, the capability of G-KANs to achieve continual learning is not a result of task correlation.

Supplementary Note 6: Comparison of computational units for implementing KANs.

As understood from the above and main text in this manuscript, the core of KAN's computation lies in basis functions. Although previous studies have proposed using lookup tables (LUTs) in digital circuits to approximate B-spline functions and reduced recursive operations[6,12], LUTs suffer from severe scalability limitations. Their size increases exponentially with the number of classes, causing a sharp increase in the requirement of transistors, and severely limiting the scalability of physical KAN. Although implementing B-spline functions with analog circuits offers certain advantages in resource utilization and potential scalability,[28] both analog and digital designs generally rely on physically separated computing and memory architectures, fundamentally limiting computational efficiency.

In comparison, GMCs inherently generate Gaussian-like functions, reducing the complexity of generating basis functions, while enabling computing in memory. More impactfully, GMC's inherent programmability and in-situ computation capabilities, are well-suited for physical KAN's implementations. Supplementary Table 2 provides a direct comparison of hardware resources, clearly demonstrating that employing GMCs in building efficient physical KAN's systems allow to significantly reduce hardware usage and energy consumption. Notably, Lozano Duarte et al. reported that the digital CMOS implementation consumed 266.735 μW of power and occupied an area of 9.111 mm^2, whereas the analog CMOS version consumed 238.5 μW and required 0.073 mm^2.[28] In contrast, the proposed GMC achieves a significantly lower power

consumption on the order of nanowatts (calculated as operating multiplication of voltage and current), with an area of merely 2.2×10^{-4} mm^2, thereby demonstrating advantages in both energy efficiency and area on a chip.

Supplementary Table 2. Comparison of different ways to implement functions

Ref.	Mode	Function	Amp. adjustability	Memory	Num. of devices	Area(mm^2)	Power (μW)
5	digital	B-Spline	No	No	LUTs* (96)	-	-
4	digital	B-Spline	No	No	LUTs* (96)	-	-
6	digital	B-Spline	No	No	-	9.111	266.735
6	analog	B-Spline	No	No	42	0.073	238.5
This work	analog	Gaussian	Yes	Yes	2	2.2×10^{-4}	$\sim 10^{-3}$

* The transistor count estimation for each 4-input LUT (4-LUT) is based on the architecture of Xilinx Virtex-4 FPGAs, where each 4-LUT can be configured as a 16-bit distributed RAM. Assuming each SRAM bit cell comprises six transistors, the total transistor count per 4-LUT can be approximated by multiplying the number of SRAM cells (16) by six, resulting in approximately 96 transistors per LUT.

Supplementary Note 7: Robustness and device uniformity evaluations of G-KANs.

As with any analog computing system, real-world implementation inevitably exists non-idealities, especially in large-scale arrays. However, the key feature of our proposed architecture is the foundation on programmable memristors, which allows the learnable coefficients of individual cells to automatically compensate during training for non-ideal effects such as current drift caused by device variations[23,29]. In detail, in-situ training can adaptively adjust network parameters to minimize the impact of unavoidable hardware non-idealities without requiring any prior knowledge of the hardware. Consequently, this approach possesses the capability to automatically modulate weights and compensate for hardware defects.[29] In deeper networks, the adaptive characteristic of in-situ training becomes particularly pronounced, as hidden neurons can minimize the impact of hardware defects on network performance.[29] Such adaptability significantly reduces the impact of analog device errors on our proposed architecture. To evaluate the impact of errors on the performance of G-KANs, two typical task types were conducted: regression and classification. In the 1D function regression task, despite the injected noise, the G-KAN even maintained a high level of fitting precision when introducing a perturbation twice the magnitude of the intrinsic case, indicating minimal performance degradation after in-situ training. To further evaluate the robustness of the G-KAN's model, the *C*v values were scaled by a factor q to simulate different levels of non-idealities. When $q = 1$, it indicates that only the intrinsic variability of the device is considered, whereas $q > 1$ implies the presence of additional external disturbances. As shown in Supplementary Fig. 16a, the RMSE

gradually increases with the factor q from 1 to 50 on the 1D function regression task. Even under the noise levels increased to 50 times the intrinsic case, the RMSE on this task remained below 0.4, demonstrating the robustness of the G-KAN architecture under severe variation conditions. Furthermore, the performance of noisy G-KAN in image classification is evaluated as well. As illustrated in Supplementary Fig. 16b, the learning curves under different noise presets indicate that the classification accuracy also remains robust under realistic noise conditions—introduced by GMCs' variability and external disturbances—particularly when the noise scaling factor $q \leq 15$.

On the other hand, the theoretical analysis of scalability limits remains an impactful research direction. One promising approach is to develop a modeling framework that accounts for device variability and analyzes how key performance metrics—such as accuracy or robustness—change with network scale. When incorporating the intrinsic non-idealities of GMCs, the G-KAN was evaluated on a more complex classification task by enlarging the architectural scale. The performance of the system is within an acceptable range when affected by non-ideal factors at the device level (Supplementary Fig. 16c).

Supplementary Note 8: Energy efficiency comparison between the G-KAN system and CMOS architectures.

This section conducts a comparison study between the GMC-based system and two NVIDIA GPUs, focusing on the energy efficiency differences among these architectures. The experiments are based on a preset network scale of KAN [784, 100, 10] (grid size = 4) for the G-KAN architecture. In the simulation, the inference latency is set to 300 ns, and the average power consumption includes both array energy (E_{array}) and peripheral circuit energy consumption (E_{perip}). Among the components, the E_{perip} mainly comes from the operational amplifier combinations used in the neuron implementation based on the differential-pair mechanism, with each output neuron equipped with three high-bandwidth, low-power operation amplifiers[30,31]. Notably, the detailed formulas for energy consumption calculation are as follows:

$$n_{\text{device}} = \left(784 \times 100 + 100 \times 10\right) \times 4 \times 2 = 635200 \tag{S9}$$

$$n_{\text{opa}} = \left(100 + 10\right) \times 3 = 330 \tag{S10}$$

$$E_{\text{array}} = 635200 \times 16.6345 \text{ nW} \times 300 \text{ ns=}3.17 \text{ nJ} \tag{S11}$$

$$E_{\text{perip}} = 330 \times 0.34 \text{ mW} \times 300 \text{ ns} = 33.66 \text{ nJ} \tag{S12}$$

$$E_{\text{total}} = E_{\text{array}} + E_{\text{perip}} = 36.83 \text{ nJ} \tag{S13}$$

Here, n_{device} represents the total number of devices in the GMC array, and n_{opa} denotes the total number of operational amplifiers used to implement the neurons. Moreover, according to the definition of energy efficiency[10,23,31,32], the computational workload must first be quantified. Since the GMC array is capable of performing the

parameterized Gaussian transformation and accumulation ($\sum A\cdot\exp\left[-\left(\frac{x-\mu}{\sigma}\right)^2\right]$) operation in one step, for a fair comparison, such operations need to be equated to the corresponding number of operations in CMOS architectures. In computer electronics, the operation $\sum A\cdot\exp\left[-\left(\frac{x-\mu}{\sigma}\right)^2\right]$ is executed step by step: $x-\mu \rightarrow \frac{x-\mu}{\sigma} \rightarrow \left(\frac{x-\mu}{\sigma}\right)^2 \rightarrow -\left(\frac{x-\mu}{\sigma}\right)^2 \rightarrow \exp\left[-\left(\frac{x-\mu}{\sigma}\right)^2\right] \rightarrow A\cdot\exp\left[-\left(\frac{x-\mu}{\sigma}\right)^2\right] \rightarrow \sum A\cdot\exp\left[-\left(\frac{x-\mu}{\sigma}\right)^2\right]$. Among these steps, the transcendental function $\exp(\cdot)$ in double precision calculations is implemented through the floating-point architecture based on the reduction-approximation-reconstruction principle, which involves a total of 29 operations.[33] Therefore, the $\sum A\cdot\exp\left[-\left(\frac{x-\mu}{\sigma}\right)^2\right]$ operation in computer hardware is equivalent to 35 operations in total. Based on this, the energy efficiency of the GMC-based system can be calculated as follows:

$$\mathrm{EE} = \frac{635200 \times 35\ \text{operations}}{36.83\ \text{nJ}} = 603.64\ \text{TOPS/W} \tag{S14}$$

Supplementary Table 3 presents the comparison results of energy efficiency, where the energy efficiency of the GPU is calculated based on the ratio of its peak throughput to thermal design power (TDP)[31].

Supplementary Table 3 Comparison of energy efficiency

Architecture	Energy Efficiency (TOPS/W)
RTX PRO 2000 Blackwell *1	7.79
RTX 2000 Ada Generation *2	2.74
This work	603.64

* It should be noted that the estimation is based on idealized assumptions and does not account for extra overhead in the general-purpose architectures.
1: https://www.nvidia.com/content/dam/en-zz/Solutions/products/workstations/professional-desktop-gpus/rtx-pro-2000/workstation-datasheet-blackwell-rtx-pro-2000-nvidia-us-4016661.pdf
2: https://www.nvidia.com/en-us/products/workstations/rtx-2000/

In general, the capability of GMCs to execute one-shot parameterized Gaussian kernels endow the system with fundamental characteristics of parallel computation and low power consumption for KAN-type inference process, which are key factors contributing to the high energy efficiency demonstrated in this work.

Supplementary Note 9: Evaluation of G-KAN on PDE and time series tasks.

(1) PDE solving task

For the partial differential equation (PDE) task[22], we considered Poisson equations with zero Dirichlet boundary conditions data. For $\Omega = [-1,1]^2$:

$$\begin{cases} u_{xx} + u_{yy} = f & \text{in } \Omega, \\ u = 0, & \text{on } \partial\Omega \end{cases} \tag{S15}$$

Using the data $f = -2\pi^2 \sin(\pi x)\sin(\pi y)$ for which the exact solution is $u = \sin(\pi x)\sin(\pi y)$. The loss function given by:

$$loss = \alpha loss_{\mathrm{i}} + loss_{\mathrm{b}} = \alpha \frac{1}{n_{\mathrm{i}}} \sum_{\mathrm{i}=1}^{n_{\mathrm{i}}} \left| u_{xx}(z_{\mathrm{i}}) + u_{yy}(z_{\mathrm{i}}) - f(z_{\mathrm{i}}) \right|^2 + \frac{1}{n_{\mathrm{b}}} \sum_{\mathrm{b}=1}^{n_{\mathrm{b}}} u^2 \tag{S16}$$

where $loss_{\mathrm{i}}$ is the interior loss, discretized and evaluated by a uniform sampling of n_{i} points $z_{\mathrm{i}} = (x_{\mathrm{i}}, y_{\mathrm{i}})$ inside the domain, and similarly $loss_{\mathrm{b}}$ is the boundary loss evaluated at n_{b} uniformly sampled points on the boundary. The parameter α balances the two loss components.

(2) Time-series forecasting task

For the time-series forecasting task, we use a chaotic system as benchmark test. It is inherently very challenging due to the positive Lyapunov exponent[34], which leads to exponential growth of separation of close trajectories so that even small errors in prediction can quickly lead to divergence of the prediction from the ground truth. we tested the system using the Mackey-Glass time series[35]:

$$\frac{\mathrm{d}x}{\mathrm{d}t} = \beta \frac{x(t-\tau)}{1 + \left[x(t-\tau)\right]^n} - \gamma x(t) \tag{S17}$$

We set the parameters to $\beta = 0.2$, $\gamma = 0.1$, $\tau = 18$, and $n = 10$. G-KAN and M-MLP were

trained to predict the next time step based on the previous 20 time-steps.

Supplementary Note 10: Analysis of error statistics.

Given that the Gaussian-like function is described by characteristic parameters such as the peak value, σ_+ and σ_-, error analysis can be conducted on the perturbations introduced in these parameters. Mathematically, these parameters can be defined as a class of variables that satisfy the following equation:

$$P^* = P(1 + KZ) \quad \text{(S18)}$$

where the P^* denotes the experimentally measured value, Z is a variable following a given distribution, and K is defined as the characteristic perturbation constant (equivalent to the coefficient of variation).[36]

Regarding whether a q-fold perturbation level is sufficient for real-world hardware, we refer to the study by Li et al[29]. In practice, in addition to the intrinsic device variability, array-level operation also suffers from perturbations introduced by peripheral circuits, such as sensing drift in the transimpedance amplifier (TIA).[29] The standard deviation of such drift has been reported to be approximately 0.3, which is still far below the case of 50-fold intrinsic variability (corresponding to $P^* \sim \mathcal{N}(P, 2500(KP)^2)$). Notably, when $q = 50$, the RMSE approximately reaches as high as 0.38. Although this represents a considerable error level for a 1D function regression task, it should be emphasized that such performance arises under significantly amplified perturbations—50 times the intrinsic device variation. The motivation for presenting this stress-test scenario was to benchmark against a M-MLP model with 15,250 weights (i.e., 152.5× larger than G-KAN with $n_c = 100$). However, this M-MLP exhibited an RMSE as high as 1.311 even under ideal conditions. Hence,

such extreme test serves merely as a reference point in the previous revised manuscript. In realistic settings, GMC arrays operate under normal device-to-device interactions, where the perturbation factor q is expected to be close to 1. Even when $q = 2$ — already far beyond the typical operational conditions—the RMSE increases only to 0.037, demonstrating that the system maintains strong robustness under practical circumstances.

Following the reasoning of Li et al., if the network maintains robust performance (measured by AI benchmark metrics) even under artificially magnified error conditions (e.g., $q = 2$) that significantly exceed the expected real-world variability, this strongly substantiates the reliability of the G-KAN in practical operation. Moreover, the underlying mechanism of neural network for classification is probabilistic decision-making, where the label with the highest probability is selected as the output.[37] Under this principle, even if the predicted probabilities are affected by internal errors, the final classification outcome does not vary continuously as RMSE does in regression tasks, but instead exhibits a certain degree of tolerance. Consequently, classification accuracy is less sensitive to perturbations. As shown in Fig. 5g of main text, even when $q = 5$, the accuracy decreases by only 0.453%. Therefore, G-KAN demonstrates strong robustness in both regression and classification tasks.

Supplementary Note 11: Circuit for implementing activation in the residual connection.

In the original design of KANs, the SiLU (or swish) activations are used in the residual connections[22], but the exponential term (e^{-x}) in SiLU(x) is challenging to implement in circuits[38]. Coincidentally, ReLU introduces a global nonlinear activation[22] similar to SiLU, and is much simpler to implement at the hardware level (see Supplementary Table 4). Hence, it is necessary to replace SiLU with ReLU for the hardware implementation of KANs. The detailed analysis is presented as follows.

Firstly, to provide a more intuitive comparison of the hardware implementation complexity between the SiLU and ReLU nonlinear activation functions, we present the complete circuit schematics of both the SiLU[38] and ReLU designs (Supplementary Fig. 20). Specifically, the construction of the SiLU circuit[38] is relatively challenging, as this circuit involves an exponential circuit (highlighted in the orange box), an external constant current source (1 A), two operational amplifiers, and a division circuit. In particular, the exponential circuit additionally requires a constant current source supplying the reference current and two operational amplifiers.[38] Under these conditions, configuring such a complex circuit merely to generate the output of a single $\mathrm{SiLU}(x)$ function is highly impractical for large-scale network integration.

On the other hand, the ReLU function ($\mathrm{ReLU}(V_\mathrm{i}) = \max\{0, V_\mathrm{i}\}$) can be implemented through a combination of the feedback amplifiers and diodes to achieve analog-level transformation (Supplementary Fig. 21a). This circuit has been validated

through Simscape Electrical simulations, and the ReLU operation is well-supported by the simulation result (Supplementary Fig. 21b and c). This approach is designed to transmit the voltage in the ReLU form and effectively determine the polarity of the input signals.

Supplementary Tabel 4. Implementation of SiLU(x) and ReLU(x)

Activation	Expression	Difficulty
SiLU(x)	$\frac{x}{1+e^{-x}}$	hard
ReLU(x)	$\max\{0, x\}$	far easier

Supplementary Note 12: Training algorithms for backpropagation

During the simulation, the networks update towards the target states based on the output results using the gradient descent algorithms (AdamW[39] or SGD[40,41]) and loss functions (L1 loss[42,43] or cross-entropy loss[44,45]). The corresponding strategies were used for different tasks including 1D regression (1D RG) and pattern recognition with (PR w/ CL) or without continual learning (PR w/o CL). All of the details are illustrated in Supplementary Table 5. Additionally, in the 1D regression task, since the grid size is expanded to 100. Specifically, the training dataset consists of five segments of Gaussian peaks ($f_i(x) = \exp[-300(x - c_i)^2]$ $(i = 1,2,3,4,5)$, where the $f_i(x)$ and c_i represent the function and the center of the i-th Gaussian peak), with each segment containing 1,000 data points.

Supplementary Table 5. Loss functions and optimizers utilized for different tasks

Task	Loss function	Optimizer
1D RG	L1 loss (MAE loss)	AdamW
PR w/o CL	Cross-entropy loss	AdamW
PR w/ CL	Cross-entropy loss	SGD
PDE	MSE loss	AdamW
Time-series forecasting	MSE loss	AdamW

The explanation of the contents in this table is as follows:

(1) L1 loss:

$$\mathcal{L}(y, \hat{y}) = \frac{1}{N}\sum_{i=1}^{N}\left|y_i - \hat{y}_i\right| \qquad \text{(S19)}$$

where the y_i and $\hat{y}_i$ are the i-th predicted value, respectively. And N denotes the

number of samples.

(2) Cross-entropy loss:

Cross-entropy loss combines softmax[14] and cross-entropy to measure the difference between the predicted values and the actual labels of a model, which can be expressed as the equations below:

$$\hat{y}_{\mathrm{i}} = \frac{\exp(z_{\mathrm{i}})}{\sum_{\mathrm{j}=1}^{\mathrm{C}} \exp(z_{\mathrm{j}})} \quad \text{(S20)}$$

$$\mathcal{L} = -\frac{1}{\mathrm{N}} \sum_{\mathrm{i}=1}^{\mathrm{N}} \sum_{\mathrm{c}=1}^{\mathrm{C}} y_{\mathrm{i,c}} \log(\hat{y}_{\mathrm{i,c}}) \quad \text{(S21)}$$

Here, z_{i} is the logit value for class i, C is the number of classes, and $\hat{y}_{\mathrm{i}}$ is the predicted probability for class i. In equation (S21), $y_{\mathrm{i,c}}$ represents the true label of the i-th sample for class c (if the sample belongs to class c, then $y_{\mathrm{i,c}} = 1$, otherwise $y_{\mathrm{i,c}} = 0$). On the other hand, $\hat{y}_{\mathrm{i,c}}$ denotes the predicted probability of the i-th sample for class c.

(3) MSE loss:

$$\mathcal{L}(y, \hat{y}) = \frac{1}{\mathrm{N}} \sum_{\mathrm{i}=1}^{\mathrm{N}} (y_{\mathrm{i}} - \hat{y}_{\mathrm{i}})^2 \quad \text{(S22)}$$

where the y_{i} and $\hat{y}_{\mathrm{i}}$ are the i-th predicted value, respectively. And N denotes the number of samples.

(4) AdamW optimizer:

For the gradient ($g_t = \nabla_{\theta} \mathcal{L}_t(\theta_{t-1})$) ($t = 1, 2, \cdots$), the update algorithm of the parameter (θ) is as follows:

$$\theta_t = \theta_{t-1} - \gamma \lambda \theta_{t-1} - \gamma \hat{m}_t / (\sqrt{\hat{v}_t^{\max}} + \epsilon) \quad \text{(S23)}$$

where the $\hat{m}_t = \frac{m_t}{1-\beta_1^t} = \frac{\beta_1 m_{t-1} + (1-\beta_1) g_t}{1-\beta_1^t}$ ($\hat{\beta}_t = 0.9$) represents the corrected result of the first moment (m_t), the $\hat{v}_t^{\max} = \max\{v_t^{\max}, \hat{v}_t\}$, represents the maximum value of the corrected second moment ($\hat{v}_t = \frac{v_t}{1-\beta_2^t} = \frac{\beta_2 v_{t-1} + (1-\beta_2) g_t^2}{1-\beta_2^t}$) ($\beta_2 = 0.999$), and ϵ is a term added to the denominator to improve numerical stability[10] ($\epsilon = 1\times 10^{-8}$ by default). In addition, γ and λ are the learning rate and weight decay, respectively.

(5) SGD optimizer:

To reduce overfitting, the SGD algorithm is used in continual learning for pattern recognition:

$$\theta_t = \theta_{t-1} - \gamma g_t - \gamma_{\mathrm{k}} \lambda \theta_{t-1} \tag{S24}$$

where the γ_{k} $(\mathrm{k} = 1, 2)$ represents the learning rate utilized for the k-th dataset.

Supplementary Figures

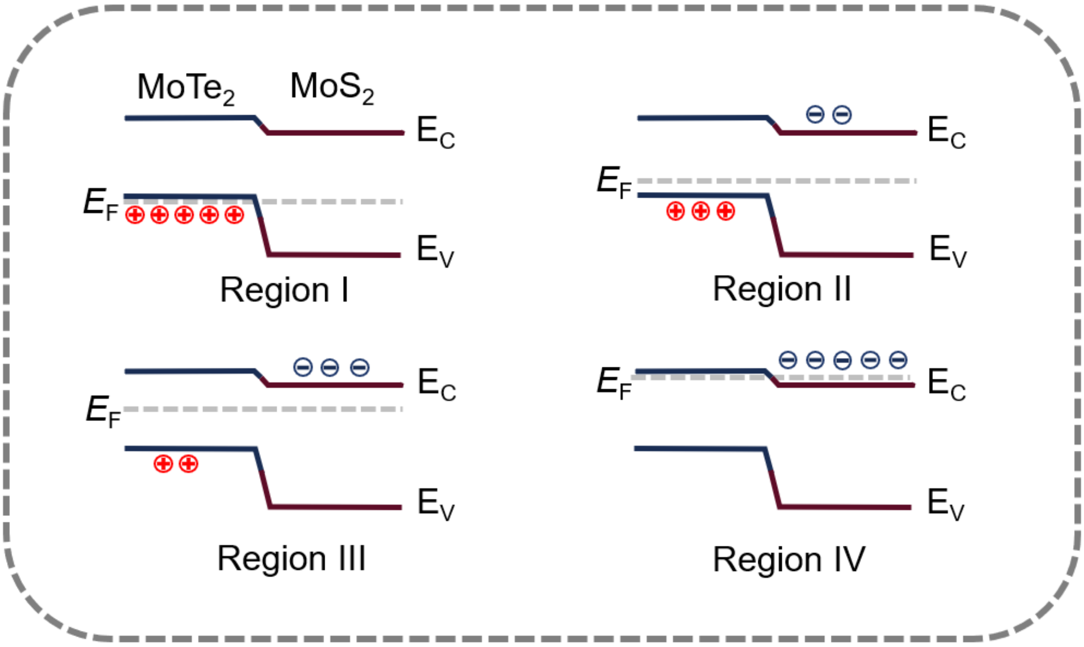

Supplementary Fig. 1 | Energy band arrangement diagram of heterojunction in the Gaussian transistor un different operating regions.

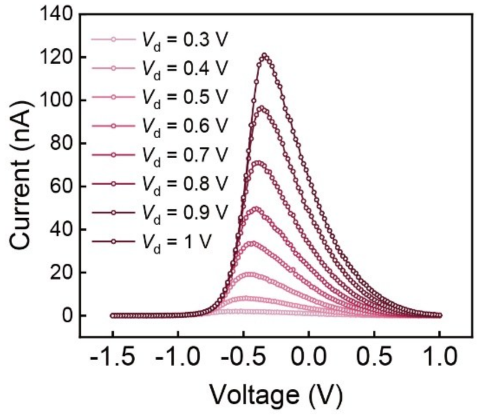

Supplementary Fig. 2 | Transfer characteristics of gauss transistors. The drain current (I_d) versus gate voltage (V_g) of the Gaussian transistor for different drain voltage (V_d).

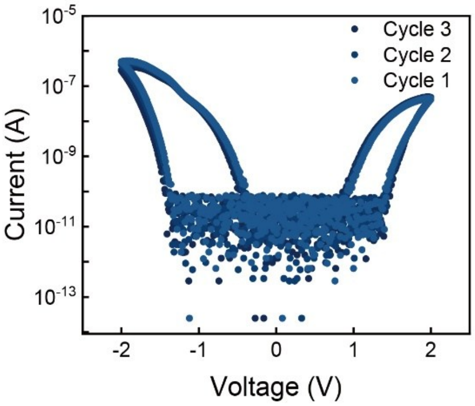

Supplementary Fig. 3 | *I-V* characteristics of the memristor while applying a sweep voltage ranging from -2 to 2 V. These curves represent the *I-V* characteristics for three cycles continuously tested in sequence.

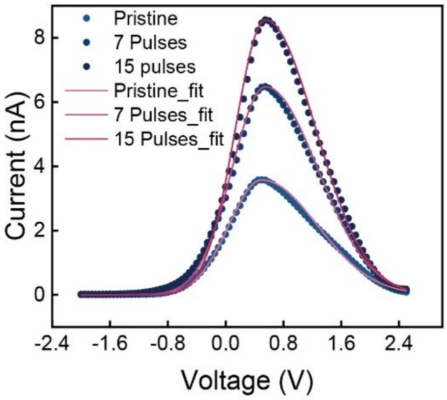

Supplementary Fig. 4 | Fitting results of the Gaussian-like transfer characteristics.

The original data and the corresponding fitting results under three different write pulses.

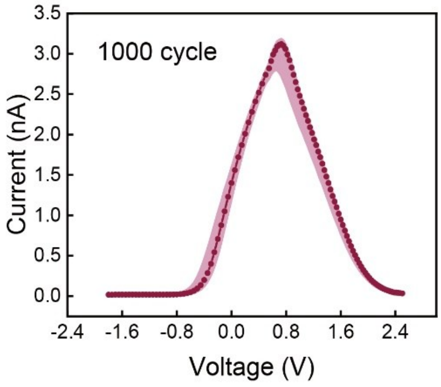

Supplementary Fig. 5 | Endurance test. Test of the transfer characteristic curve for 1000 cycles of the same GMC unit, among which the wine red color represents the output of the 500th cycle.

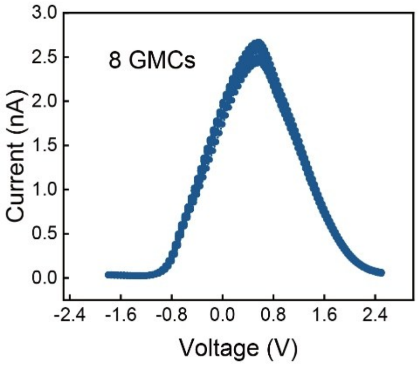

Supplementary Fig. 6 | The transfer characteristic curves with 8 different GMCs.

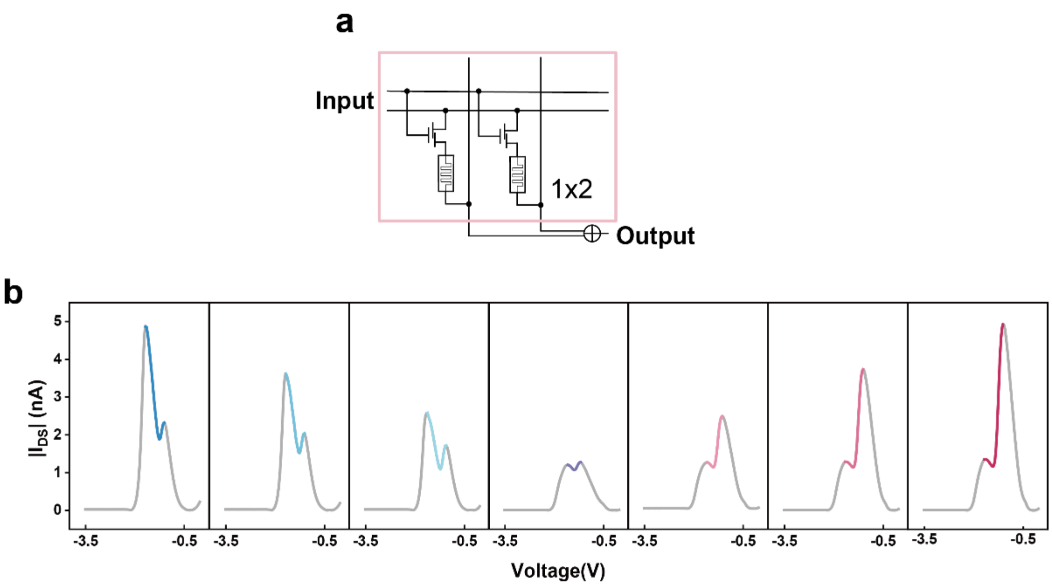

Supplementary Fig. 7 | 1×2 array achieving a learnable activation function. a A parallel circuit of two GMCs. **b** Curves of current versus gate input voltage for two parallel GMC units after applying 2, 4, and 6 pulses to individual GMCs, and the constructed activation functions.

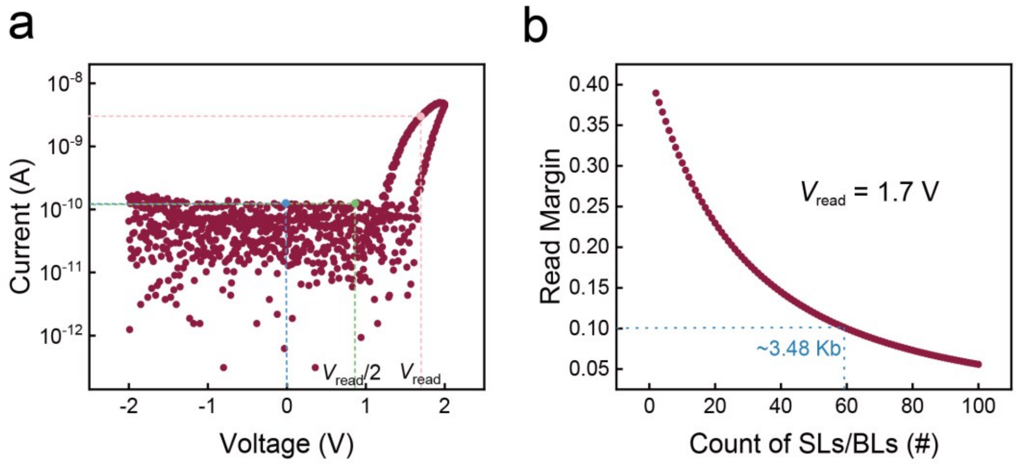

Supplementary Fig. 8 | Statistical analysis of the array scalability. a The self-rectifying *I*-*V* characteristics of GMCs. **b** The scalability analysis based on the proposed GMCs with over 3.48 kb scale.

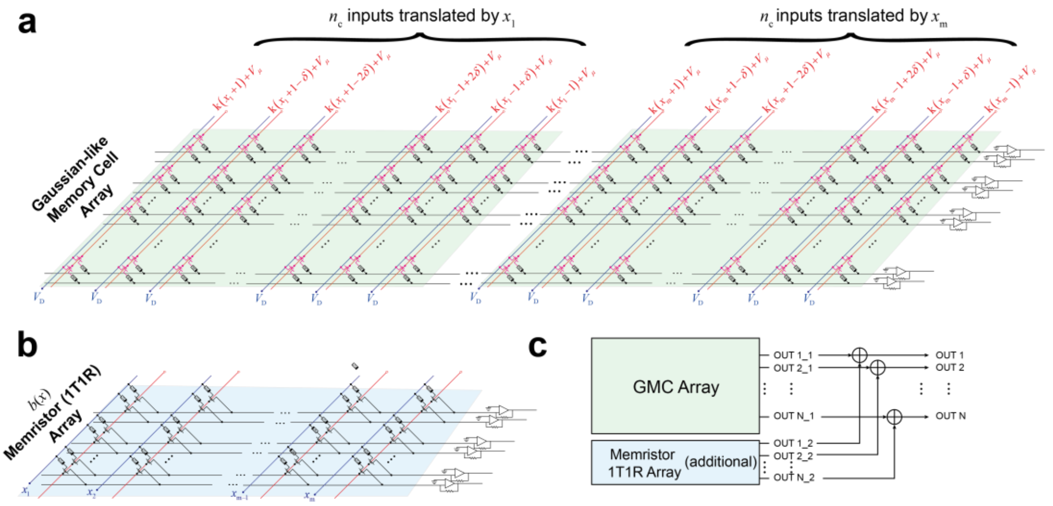

Supplementary Fig. 9 | Schematic diagram of the extended G-KAN architecture with residual connections. a GMC array part. Each green block represents the combination of n_c Gaussian-like basis functions expanded from an input variable (x). **b** A 1T1R-standard integrated memristor array is employed to process the additional operations of the residual-connection part. **c** The GMC array and the $b(x)$ array are directly superimposed element-wise along the output vector to generate the final output.

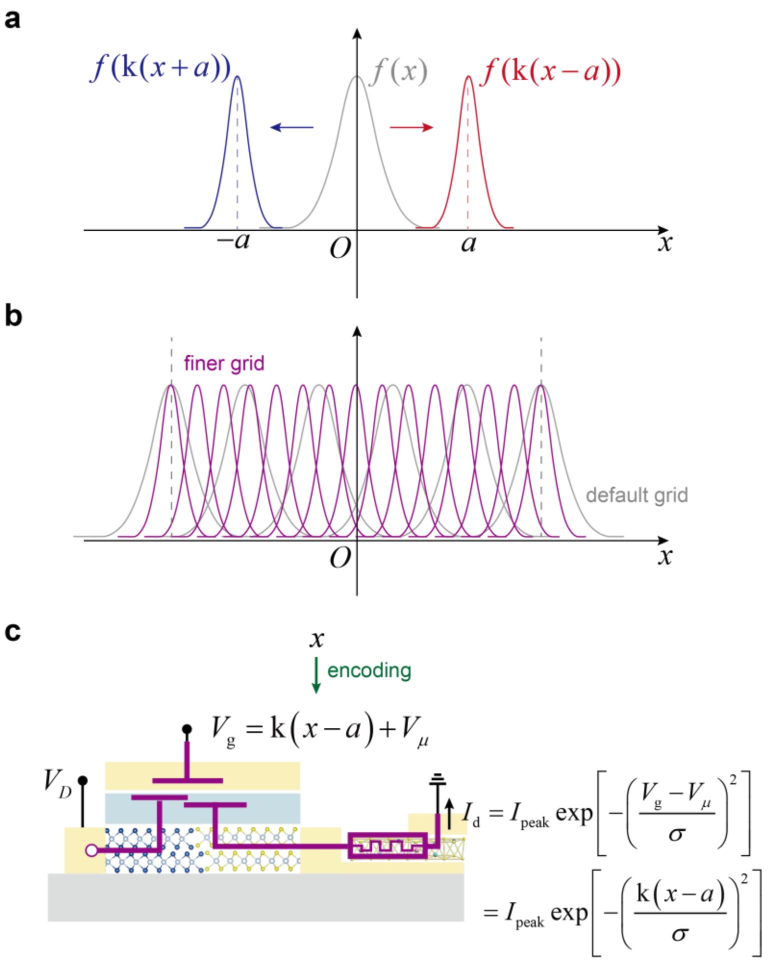

Supplementary Fig. 10 | Function translation and device mapping. a Function translation induced by variable bias. **b** Function contraction induced by gain on the independent variable for finer-grid adaption. **c** The input signal (x) is embedded in the voltage applied to the gate electrode, and the mapped functional relationship is established through the output characteristics of the GMC: $f(x) = \exp\left[-\left(\frac{x-a}{\sigma}\right)^2\right]$.

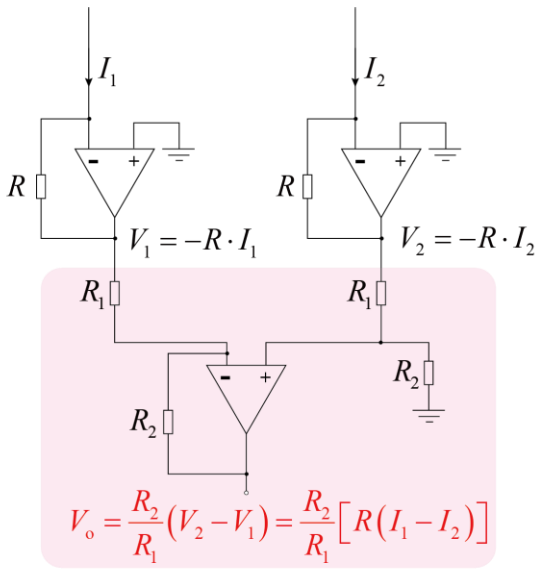

Supplementary Fig. 11 | An op-amp in the 'Subtractor Configuration', used to perform the differential operation of the voltages.

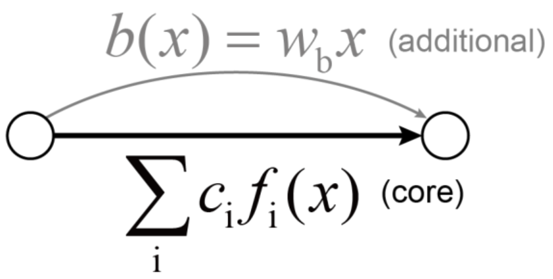

Supplementary Fig. 12 | Additional residual connection. Information transfer between nodes—residual connection ($b(x)$) can be added on the basis function combination ($\sum_i c_i f_i(x)$).

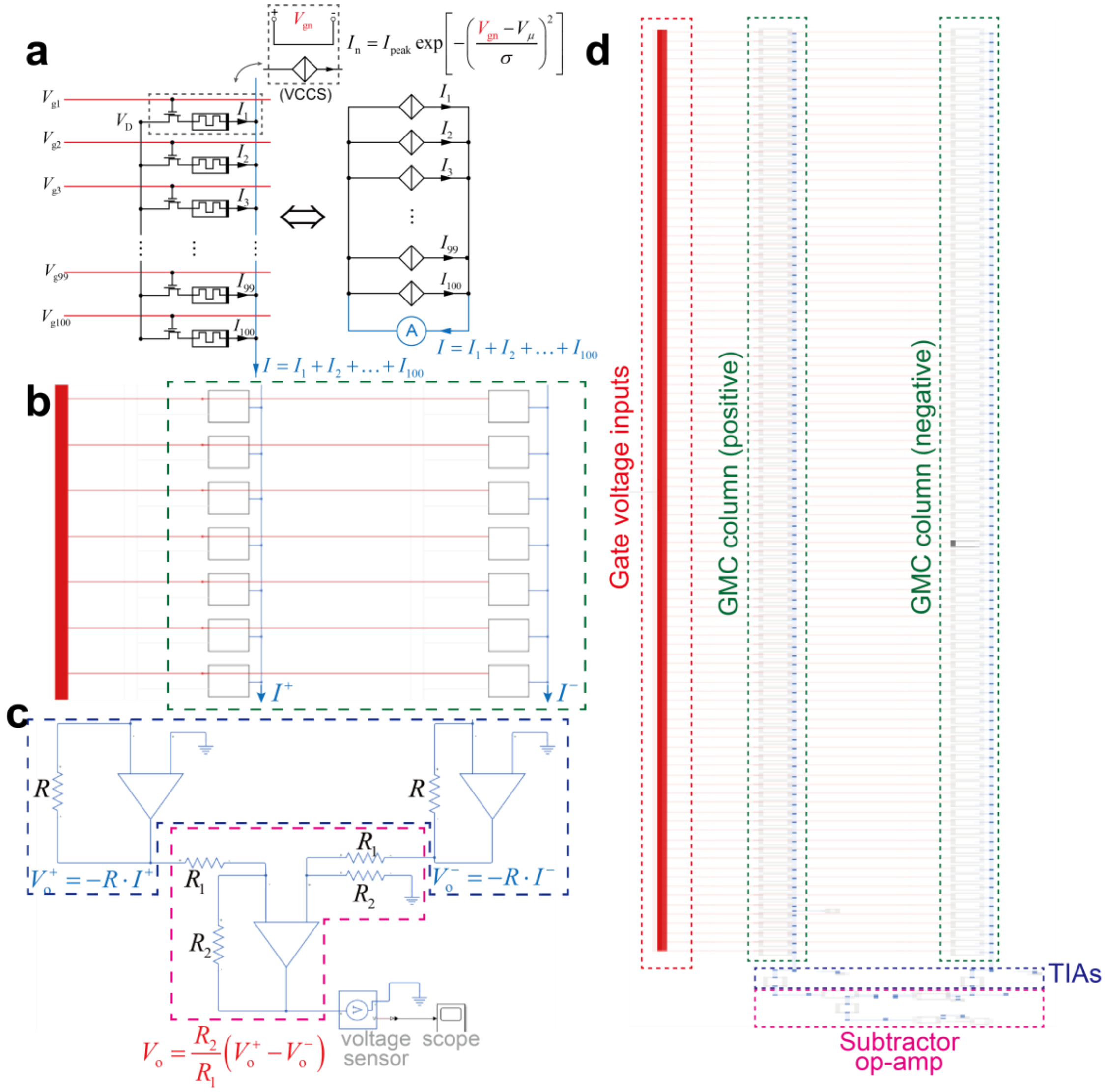

Supplementary Fig. 13 | GMC array constructed in the Simscape Electrical platform. a VCCS equivalent of the GMC branch and the parallel equivalent circuit of the GMC array. **b** Partial view of the crossbar circuit of GMCs. **c** TIAs used for voltage-mode output, while the op-amp for differential operation. **d** Demonstration of the GMC array with 100 basis functions (200 GMCs). Furthermore, the output voltages of a pair of TIAs are processed through an op-amp to perform an amplified differential operation to produce the final output of the G-KAN's network computation. The output waveform is then observed using a 'Voltage Sensor' and a 'Scope' block.

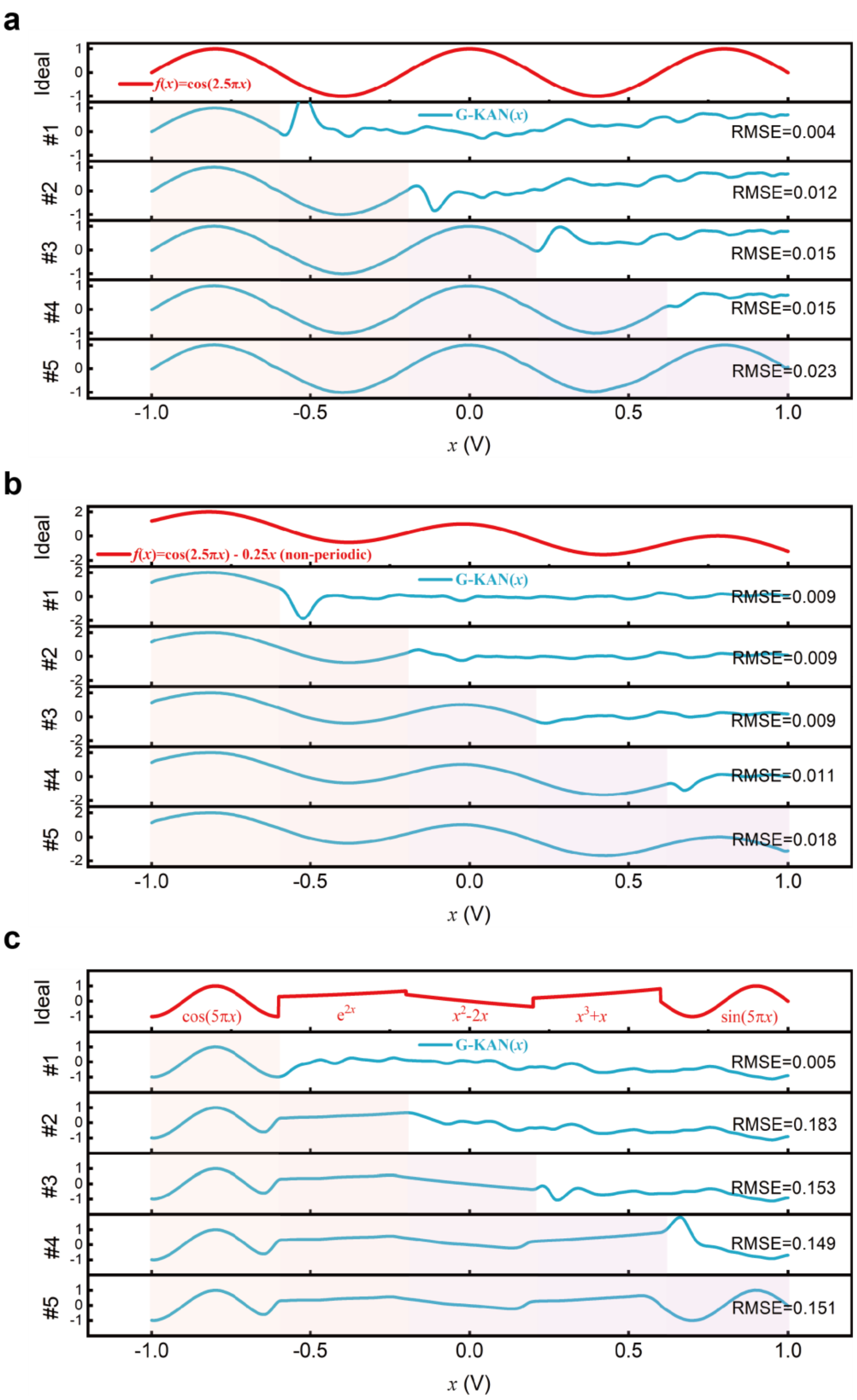

Supplementary Fig. 14 | Fitting performance of the G-KAN on non-repetitive function datasets. Models are trained by **a** A single non-Gaussian periodic function. **b** A non-periodic function. **c** A piecewise discontinuous function.

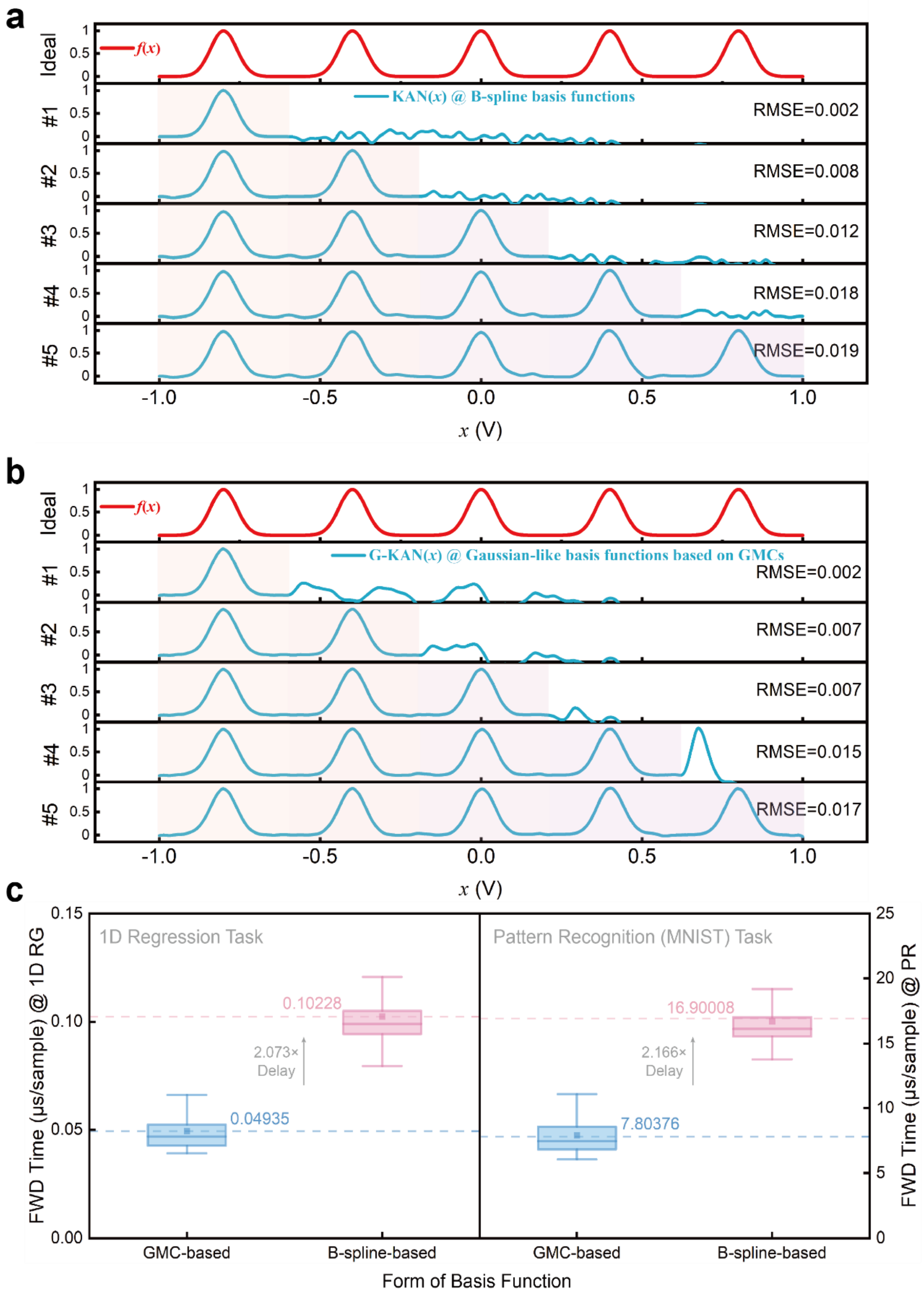

Supplementary Fig. 15 | The performance comparison of KANs with different basis functions. a The performance of a KAN when using B-splines. **b** The performance of a G-KAN based on GMCs. **c** Statistics on the time consumption of basis functions based on GMCs or B-splines in KANs. Note that in each box plot, the upper

and lower boundaries of the box represent the upper and lower quartiles, the whiskers indicate the maximum and minimum values, the line inside the box represents the median, and the points represent the mean.

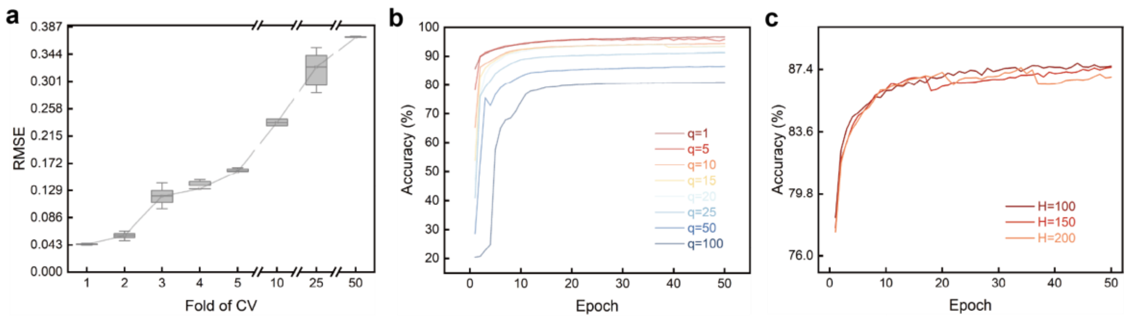

Supplementary Fig. 16 | Robustness evaluation of the G-KAN under non-idealities and increasing architectural scalability. a 1D function regression performance under variable coefficients of variation. **b** Performance of G-KAN ([784, 64, 10], $n_c = 4$) on the pattern recognition task (MNIST) with various Cvs of device inconsistency introduced. **c** Learning curves recorded under the incorporation of GMCs' non-idealities as scalability increases (G-KAN [784, H, 10] on Fashion MNIST dataset).

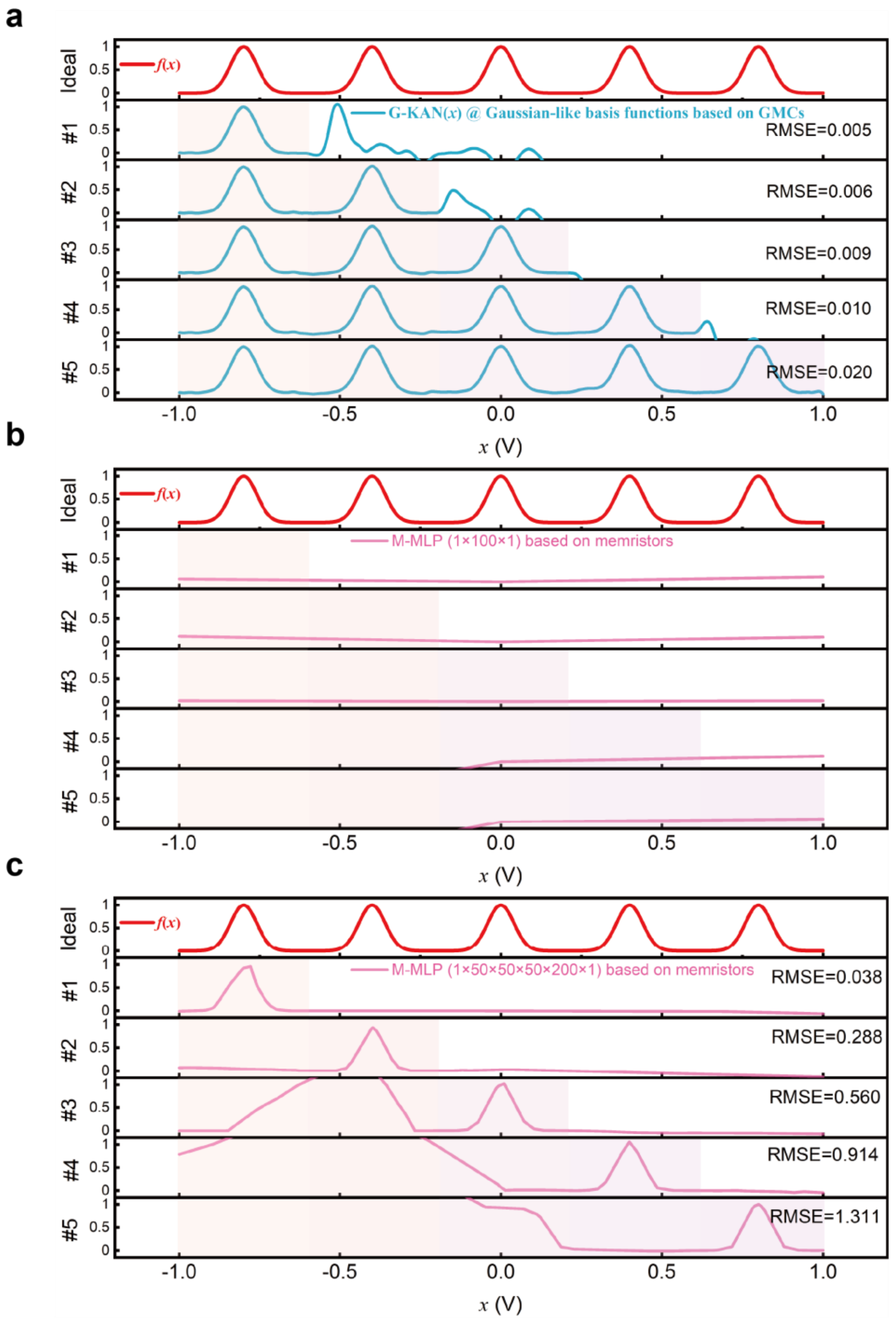

Supplementary Fig. 17 | The continual learning validations of the G-KAN and M-MLP on 1D RG tasks. a Results of G-KAN ([1, 1], n_c = 100). **b** Results of M-MLP [1, 100, 1], showing no successful fitting. **c** Results of M-MLP [1, 50, 50, 50, 200, 1], exhibiting very severe catastrophic forgetting.

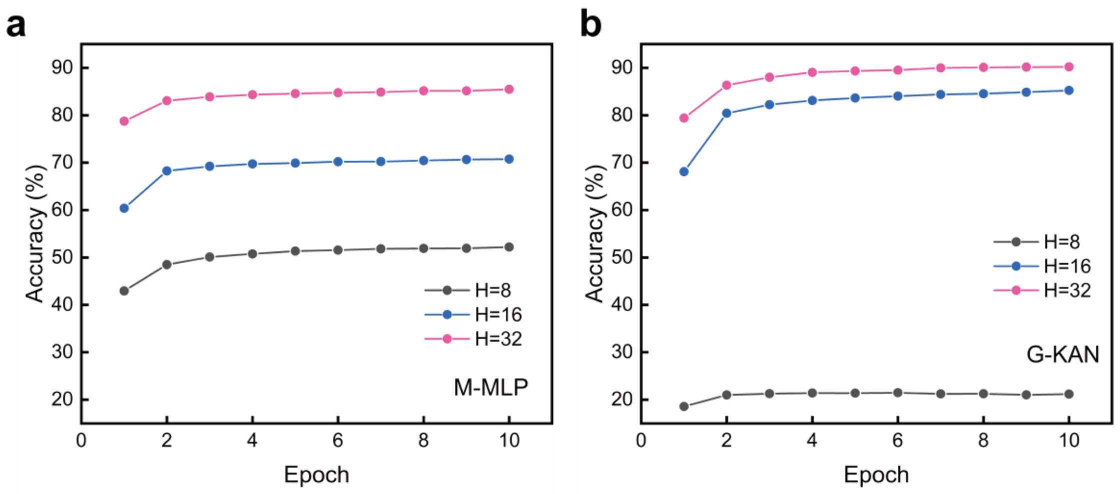

Supplementary Fig. 18 | Evaluating the neural scaling laws by adjusting different number of hidden layer nodes (H) (each network size is [784, H, 10]). a Case of M-MLP. **b** Case of G-KAN. As H increases from 8 to 16, the model performance improves in a faster manner, validating the characteristics of the faster scaling laws of KANs.

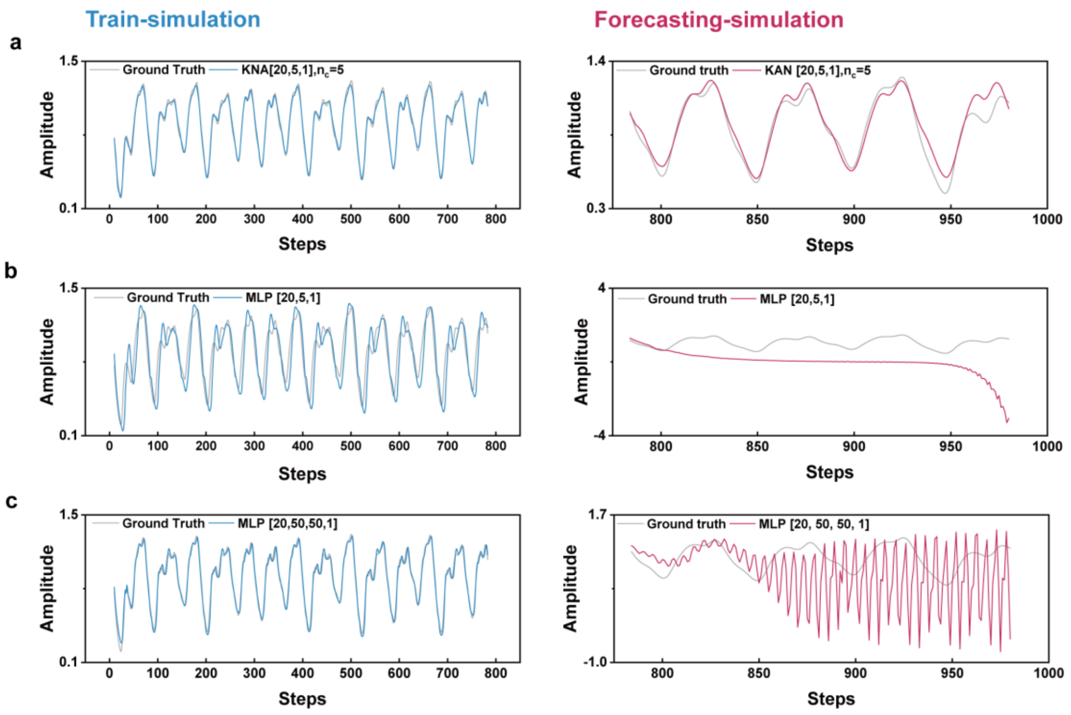

Supplementary Fig. 19 | Time-series forecasting training and forecasting results of G-KAN and M-MLP. a The structure of G-KAN is [20, 5, 1], $n_c = 5$. The structures of M-MLP are respectively **b** [20, 5, 1] and **c** [20, 50, 50, 1].

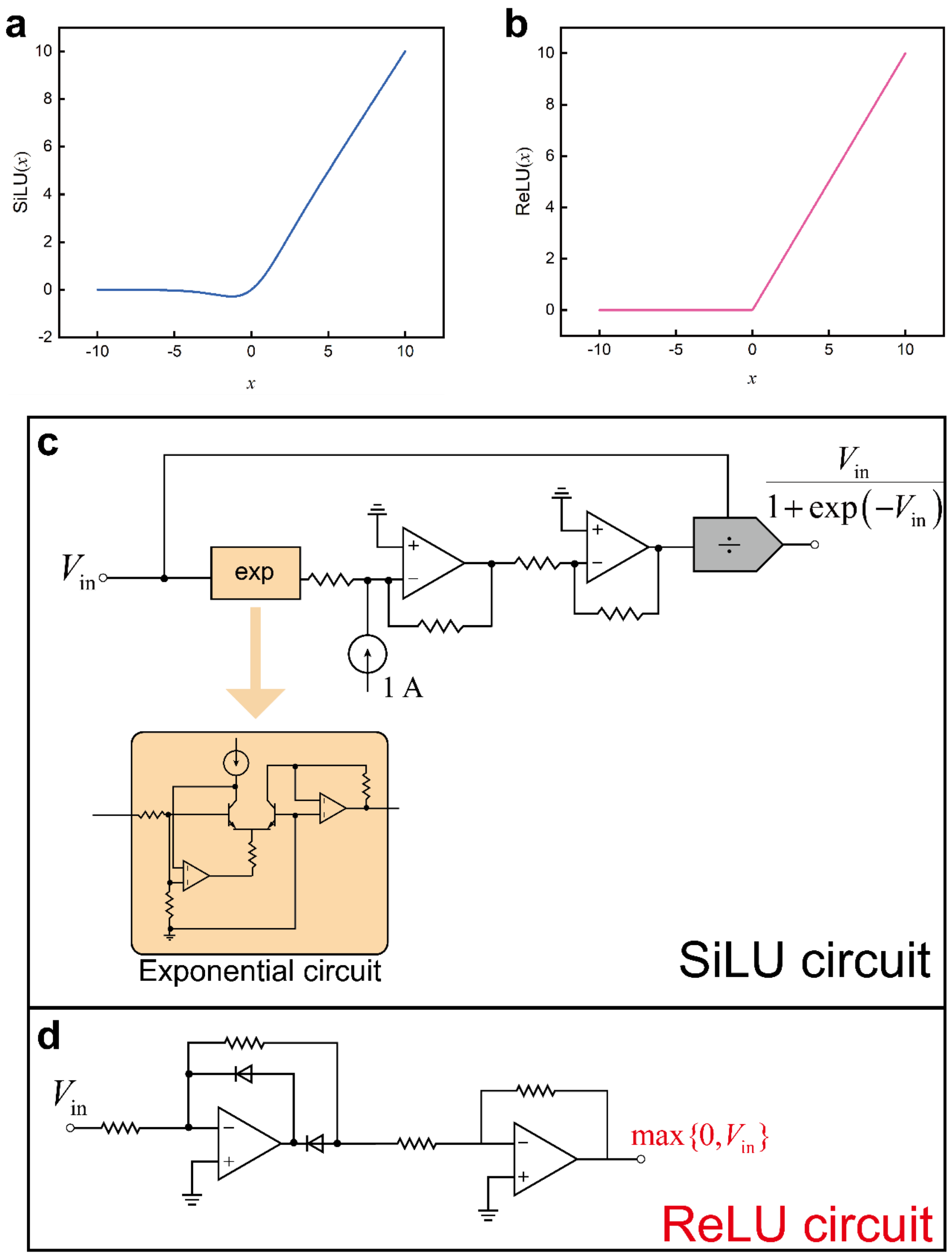

Supplementary Fig. 20 | Hardware implementation of SiLU and ReLU functions. **a** Graph of $\mathrm{SiLU}(x)$. **b** Graph of $\mathrm{ReLU}(x)$. **c** Schematic of SiLU circuit. **d** Schematic of ReLU circuit.

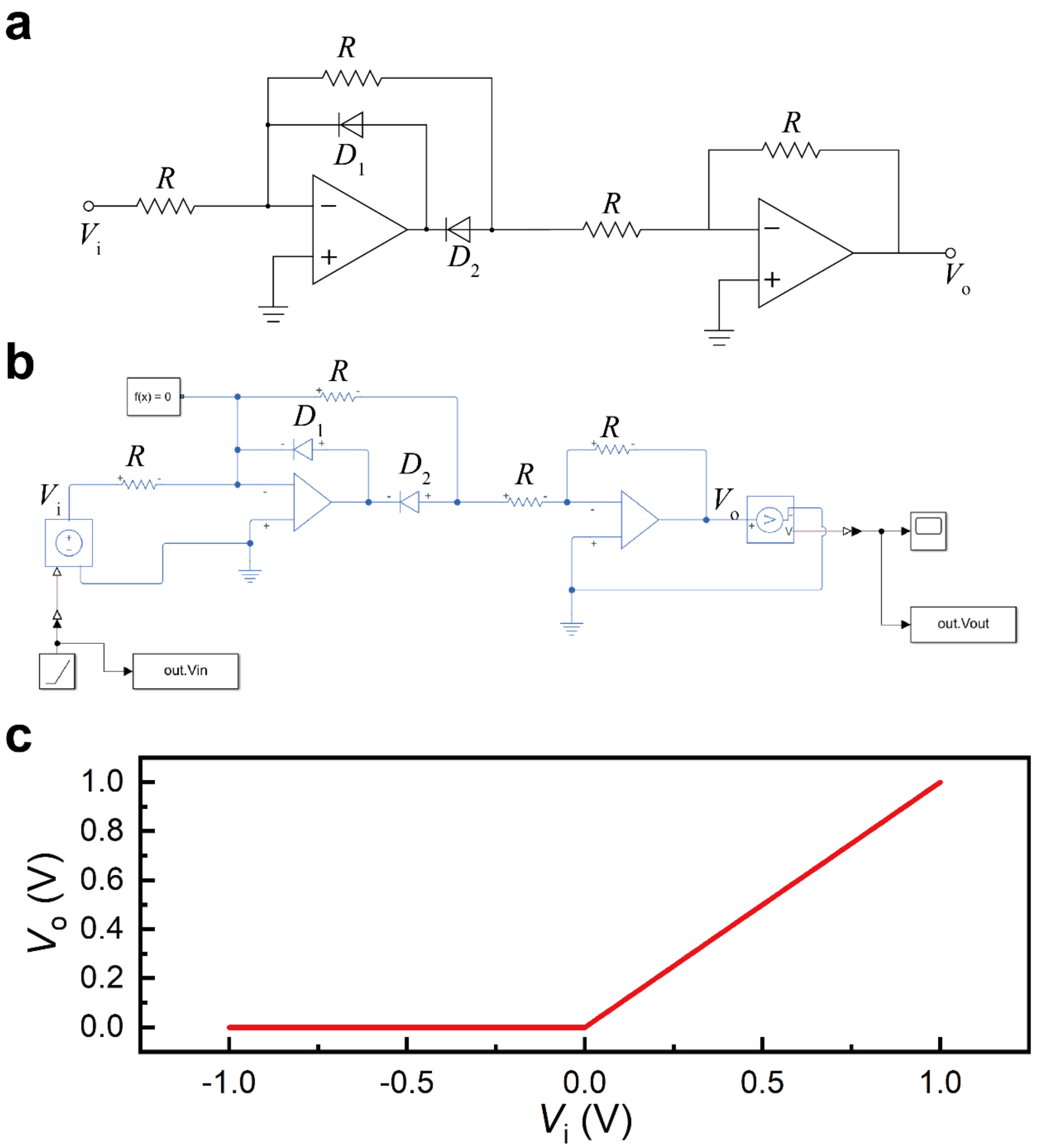

Supplementary Fig. 21 | Rectifying linear unit (ReLU) circuit. a Circuit schematic. V_i and V_o represent the input and output voltages of the ReLU function circuit module, respectively. **b** Circuit built within the Simscape electrical simulation platform. For the configuration, V_i is swept from -1 V to 1 V using a controlled voltage source, and the V_o is measured using a voltage sensor. **c** V_o-V_i characteristic curve, which is consistent with the numerical simulation of ReLU.

References

1 Vijaykumar, N. *et al.* A framework for accelerating bottlenecks in GPU execution with assist warps. Preprint at https://arxiv.org/abs/1602.01348 (2016).

2 Maurya, A. K., Rafique, M. M., Cappello, F. & Nicolae, B. MLP-Offload: Multi-level, multi-path offloading for LLM pre-training to break the GPU memory wall. In *Proceedings of the International Conference for High Performance Computing, Networking, Storage and Analysis*, 1381-1394 (ACM, 2025).

3 Ansari, M. Q. & Ansari, M. Q. Accelerating Matrix Multiplication: A Performance Comparison Between Multi-Core CPU and GPU. Preprint at https://arxiv.org/abs/2507.19723 (2025).

4 Ho, K., Zhao, H., Jog, A. & Mohanty, S. Improving GPU throughput through parallel execution using tensor cores and CUDA cores. In *2022 IEEE Computer Society Annual Symposium on VLSI (ISVLSI)*, 223-228 (IEEE, 2022).

5 Driss, S. B., Soua, M., Kachouri, R. & Akil, M. A comparison study between MLP and convolutional neural network models for character recognition. In *Real-Time Image and Video Processing 2017*, 32–42 (SPIE, 2017).

6 Le, T. X. H. *et al*. Exploring the limitations of Kolmogorov-Arnold networks in classification: Insights to software training and hardware implementation. In *2024 Twelfth International Symposium on Computing and Networking Workshops* (CANDARW), 110-116 (IEEE, 2024).

7 Singh, G. *et al.* A review of near-memory computing architectures: Opportunities and challenges. In *2018 21st Euromicro Conference on Digital System Design* (DSD), 608-617 (IEEE, 2018).

8 Shymkovych, V., Telenyk, S. & Kravets, P. Hardware implementation of radial-basis neural networks with Gaussian activation functions on FPGA. *Neural Comput. Appl.* **33**, 9467-9479 (2021).

9 Sudarshan, C., Manea, P. & Strachan, J. P. A Kolmogorov–Arnold Compute-in-Memory (KA-CIM) Hardware Accelerator with High Energy Efficiency and Flexibility. Preprint at https://www.researchsquare.com/article/rs-5804189/v1 (2025).

10 Aguirre, F. *et al.* Hardware implementation of memristor-based artificial neural networks. *Nat. Commun.* **15**, 1974 (2024).

11 Cintra, R. *et al.* Gaussian kernel approximations require only bit-shifts. *Information* **15**, 618 (2024).

12 Huang, W.-H. *et al.* Hardware acceleration of Kolmogorov-Arnold network (KAN) for lightweight edge inference. In *Proceedings of the 30th Asia and South Pacific Design Automation Conference*, 693-699 (2025).

13 Gungor, M. Optimizing the use of different memory types on modern FPGAs. Doctoral dissertation, Northeastern University (2024).

14 Alavani, G., Desai, J., Saha, S. & Sarkar, S. Program analysis and machine learning–based approach to predict power consumption of cuda kernel. *ACM Trans. Model. Perform. Eval. Comput. Syst.* **8**, 1-24 (2023).

15 Lam, D. & Wunsch, D. Unsupervised feature learning classification with radial

basis function extreme learning machine using graphic processors. *IEEE Trans. Cybern.* **47**, 224-231 (2016).
16 Wang, Y. E., Wei, G.-Y. & Brooks, D. Benchmarking TPU, GPU, and CPU platforms for deep learning. Preprint at https://arxiv.org/abs/1907.10701 (2019).
17 Xu, W. et al. ScaleDNN: Data movement aware DNN training on multi-GPU. In *2021 IEEE/ACM International Conference on Computer Aided Design* (ICCAD), 1–9 (IEEE, 2021).
18 Lustig, D. & Martonosi, M. Reducing GPU offload latency via fine-grained CPU-GPU synchronization. In *2013 IEEE 19th International Symposium on High Performance Computer Architecture* (HPCA), 354-365 (IEEE, 2013).
19 Dev, K. & Reda, S. Scheduling challenges and opportunities in integrated CPU+GPU processors. In *Proceedings of the 14th ACM/IEEE Symposium on Embedded Systems for Real-Time Multimedia*, 78-83 (2025).
20 Feng, W.-c. & Xiao, S. To GPU synchronize or not GPU synchronize? In *2010 IEEE International Symposium on Circuits and Systems* (ISCAS), 3801-3804 (IEEE, 2010).
21 Ojika, D. *et al.* Addressing the memory bottleneck in AI model training. Preprint at https://arxiv.org/abs/2003.08732 (2020).
22 Liu, Z. *et al.* KAN: Kolmogorov-Arnold Networks. Preprint at https://arxiv.org/abs/2404.19756 (2024).
23 Yao, P. *et al.* Fully hardware-implemented memristor convolutional neural network. *Nature* **577**, 641-646 (2020).
24 Zhang, G. *et al.* Self-rectifying memristors with high rectification ratio for attack-resilient autonomous driving systems. *Nat. Commun.* **16**, 5759 (2025).
25 Jeon, K. *et al.* Purely self-rectifying memristor-based passive crossbar array for artificial neural network accelerators. *Nat. Commun.* **15**, 129 (2024).
26 Ren, S.-G. *et al.* Pt/Al 2 O 3/TaO X/Ta self-rectifying memristor with record-low operation current (< 2 pA), low power (fJ), and high scalability. *IEEE Trans. Electron Devices* **69**, 838-842 (2021).
27 Hara, K., Saito, D. & Shouno, H. Analysis of function of rectified linear unit used in deep learning. In *2015 International Joint Conference on Neural Networks (IJCNN)*, 1-8 (IEEE, 2015).
28 Duarte, P. C. L., Dube, A., Zervakis, G., Tahoori, M. & Nassif, S. Function approximation using analog building blocks in flexible electronics. In *2025 26th International Symposium on Quality Electronic Design* (ISQED), 1-7 (IEEE, 2025).
29 Li, C. *et al.* Efficient and self-adaptive in-situ learning in multilayer memristor neural networks. *Nat. Commun.* **9**, 2385 (2018).
30 Atef, A., Atef, M., Abbas, M. & Khaled, E. E. M. High-sensitivity regulated inverter cascode transimpedance amplifier for near infrared spectroscopy. In *2016 Fourth International Japan-Egypt Conference on Electronics, Communications and Computers* (JEC-ECC), 99-102 (IEEE, 2016).
31 Lin, P. *et al.* Three-dimensional memristor circuits as complex neural networks. *Nat. Electron.* **3**, 225-232 (2020).

32 Zhang, W. *et al.* Edge learning using a fully integrated neuro-inspired memristor chip. *Science* **381**, 1205-1211 (2023).
33 Harrison, J., Kubaska, T., Story, S. & Tang, P. The Computation of Transcendental Functions on the IA-64. *Intel Technology Journal Q4*, 50-56 (1999).
34 Frazier, C. & Kockelman, K. M. Chaos theory and transportation systems: Instructive example. *Transp. Res. Rec.* **1897**, 9-17 (2004).
35 Mackey, M. C. & Glass, L. Oscillation and chaos in physiological control systems. *Science* **197**, 287-289 (1977).
36 Lee, C. *et al.* Highly parallel and ultra-low-power probabilistic reasoning with programmable gaussian-like memory transistors. *Nat. Commun.* **15**, 2439 (2024).
37 Bishop, C. M. & Nasrabadi, N. M. *Pattern Recognition and Machine Learning*, Vol. 4 (Springer, 2006).
38 Yang, C., Wang, X. & Zeng, Z. Full-circuit implementation of transformer network based on memristor. *IEEE Trans. Circuits Syst. I Regul. Pap.* **69**, 1395-1407 (2022).
39 Loshchilov, I. & Hutter, F. Decoupled weight decay regularization. Preprint at https://arxiv.org/abs/1711.05101 (2017).
40 Bottou, L. Large-scale machine learning with stochastic gradient descent. In *Proceedings of COMPSTAT'2010: 19th International Conference on Computational Statistics*, Paris, France, August 22–27, 2010, Keynote, Invited and Contributed Papers, 177-186 (Springer, 2010).
41 LeCun, Y., Bottou, L., Bengio, Y. & Haffner, P. Gradient-based learning applied to document recognition. *Proc. IEEE* **86**, 2278-2324 (1998).
42 Draper, N. *Applied regression analysis*. (McGraw-Hill. Inc, 1998).
43 Pesme, S. & Flammarion, N. Online robust regression via sgd on the l1 loss. *Adv. Neural Inf. Process. Syst.* **33**, 2540-2552 (2020).
44 Goodfellow, I., Bengio, Y., Courville, A. & Bengio, Y. *Deep Learning*, Vol. 1 (MIT Press, Cambridge, 2016).
45 Shannon, C. E. A mathematical theory of communication. *Bell Syst. Tech. J.* **27**, 379-423 (1948).
